# Atomic-scale viscoplasticity mechanisms revealed in high ductility metallic glass films

**DOI:** 10.1038/s41598-019-49910-7

**Published:** 2019-09-17

**Authors:** Hosni Idrissi, Matteo Ghidelli, Armand Béché, Stuart Turner, Sébastien Gravier, Jean-Jacques Blandin, Jean-Pierre Raskin, Dominique Schryvers, Thomas Pardoen

**Affiliations:** 1 Institute of Mechanics, Materials and Civil Engineering, UCLouvain, B-1348 Louvain-la-Neuve, Belgium; 2 EMAT, University of Antwerp, Groenenborgerlaan 171, B-2020 Antwerp, Belgium; 3 Science and engineering of materials and processes, SIMaP, Université de Grenoble/CNRS, UJF/Grenoble INP, BP46, 38402 Saint-Martin d’Hères, France; 4 Institute of information and communication technologies, electronics and applied mathematics, ICTEAM, UCLouvain, B-1348 Louvain-la-Neuve, Belgium

**Keywords:** Condensed-matter physics, Nanoscale materials, Structural materials

## Abstract

The fundamental plasticity mechanisms in thin freestanding Zr_65_Ni_35_ metallic glass films are investigated in order to unravel the origin of an outstanding strength/ductility balance. The deformation process is homogenous until fracture with no evidence of catastrophic shear banding. The creep/relaxation behaviour of the films was characterized by on-chip tensile testing, revealing an activation volume in the range 100–200 Å^3^. Advanced high-resolution transmission electron microscopy imaging and spectroscopy exhibit a very fine glassy nanostructure with well-defined dense Ni-rich clusters embedded in Zr-rich clusters of lower atomic density and a ~2–3 nm characteristic length scale. Nanobeam electron diffraction analysis reveals that the accumulation of plastic deformation at room-temperature correlates with monotonously increasing disruption of the local atomic order. These results provide experimental evidences of the dynamics of shear transformation zones activation in metallic glasses. The impact of the nanoscale structural heterogeneities on the mechanical properties including the rate dependent behaviour is discussed, shedding new light on the governing plasticity mechanisms in metallic glasses with initially heterogeneous atomic arrangement.

## Introduction

Bulk metallic glasses (BMGs) exhibit some outstanding mechanical properties involving high fracture strength, up to 3 GPa for Fe based BMGs, and high elastic strains arising from the liquid-like atomic structure with no grain boundaries, dislocations, and phase segregations, contrary to crystalline materials^1,2^. The performances of MGs include also superior corrosion resistance in harsh environments, interesting ferromagnetic properties and potential biocompatibility. However, the early occurrence at room temperature (RT) of shear band instabilities during plastic deformation of BMGs leads to a lack of ductility, thus drastically undercutting potential uses in structural applications^3^. Recently, various methods have been used to improve the RT plasticity of MGs by the production of a large amount of free volume or of excess free energy^4–6^, the introduction of nanocrystalline inhomogeneity^7,8^, the generation of a distribution of clusters with icosahedral short-range order (ISRO)^9^, the introduction of minor alloying elements^10^ and the control of nanoscale phase separation^11^. As a result, the MGs processed based on such strategies show enhanced mechanical properties at RT. However, the correlation between different atomic structuring induced by composition variation and by different processing histories, and their mechanical properties is still not very clear. Therefore, a major challenge in the field is to clarify the structural differences in these seemingly structure-less materials and to establish a causal link between the local arrangements, atomistic deformation mechanisms and macroscopic mechanical properties^12–14^.

Recent reports have also shown that the brittle-like behaviour is mitigated when the sample size is reduced down to the sub-micron scale with the suppression of catastrophic shear banding. Such behaviour has been predicted by molecular dynamic (MD) simulations^15^ and experimentally demonstrated using compression (or tension) test on micropillars^16–18^, tensile tests on long submicrometer thick ribbons^19^ or *in-situ* scanning and transmission electron microscopy (SEM and TEM) tensile tests^20–22^. This discovery has opened avenues to study mechanical size effects in thin metallic glass films (TMGFs) with potential impact on a variety of applications in micro-electro-mechanical-systems (MEMS) technology or surface coatings in harsh environments^15^. However, despite extensive research over recent years, the origin of the mechanical size effect in TMGFs is not fully unravelled yet and it can result either from geometric confinement or from a change of atomic arrangement in the films as described earlier. Indeed, the high energy and large cooling rates often involved in the sputter deposition of TMGFs might lead to heterogeneous atom distributions compared with the slowly quenched BMGs. A few recent studies in the literature have pointed out the presence of nanoscale structural and mechanical heterogeneities in TMGFs with cluster type structure^23^ along the lines discussed above. However, detailed experimental evidences of the nature of such heterogeneities including local variation of composition, atomic density and local atomic order can hardly be found in the literature^24^. Furthermore, as for BMGs, direct experimental observation of the effect of the nanoscale heterogeneities on the elementary plasticity mechanisms and the link with the formation or not of catastrophic shear bands and the resulting size effect in TMGFs remains elusive in the literature.

There is a large consensus in the literature that the elementary process controlling the macroscopic deformation of MGs is the activation, through dominantly shear-type distortion, of very localized regions (1–2 nm) called “shear transformation zones” (STZs)^25^. Significant simulation efforts (mainly MD simulations) have been devoted to explore the STZ intrinsic nature and behaviour and to connect the collective activation of STZs to the local structures and to the shear banding process in metallic glasses. Special attention has been paid to atomic-level features, i.e. short-range order (SRO) or medium-range order (MRO) of structures^13,14,26–31^, free volume^28,31,32^, and atomic-level stresses^13,33^. The stability of pentagon-rich clusters in the glassy state especially for full icosahedra plays a key role in slowing down the dynamics^34^, forming an extended and stronger elastic backbone resisting local shear deformation^14,27,28,35,36^, or showing lower strain energy^13^. STZ activation in MGs preferentially occurs at localized soft spots. Geometrically unfavoured motifs constitute the most flexible local environment that encourages soft modes and high propensity for shear transformations^37^. Free volume or local excess of free energy increases preferentially within strain localization regions or shear bands upon compression^28^, tension^31^, or shearing^32^. Very recently, Ma and co-workers introduced the “flexibility” concept to characterize the amorphous structure. The flexibility index characterizes the capacity for bond switching which is needed to mediate the local shear transformation. This concept is very generic and can be used to explain a number of effects, with enhanced flexibility directly translating into an improved ductility^38^. Nevertheless, MD simulation methods have their inherent limitations, for instance in terms of time scales that can be addressed and one remains interested in direct or indirect experimental proofs for the mechanisms discussed before.

Very recently, Ghidelli *et al*. deformed freestanding Zr_65_Ni_35_ (% at.) TMGFs in uniaxial tension using an on-chip test tensile method^19^. The authors showed that the films exhibit outstanding strength/ductility balance without the occurrence of catastrophic shear bands^19^. Significant extrinsic (i.e. sample dimensions) size effects on the strength and fracture strain were observed. However, several questions remain regarding the influence of the microstructure on the small-scale plasticity mechanisms as well as on the relationship between these mechanisms and the size dependent mechanical response including the rate dependent behaviour. The relaxation/creep viscoplastic response was not reported by Ghidelli *et al*.^19^. The analysis of rate dependent effects through transition state theory offers a way to determine the activation volume and to get indirect information on the atomistic deformation mechanisms. In addition, direct experimental observations of the STZs, of their evolution under an external applied stress and of their relationship with the structural and chemical heterogeneities in MGs are needed to go beyond empirical conjectures. Such information is still missing in the literature mainly because of the complexity associated to the characterization of amorphous microstructures and of their evolution under plastic deformation. In this context, nanobeam electron diffraction (NBED) is adapted to track the atomic scale plasticity mechanisms in MGs. Recently, Hirata *et al*., using NBED in TEM, successfully aligned and focused a coherent electron beam to a diameter as small as 0.36 nm^39^. This allowed the first measurement of electron diffraction patterns from individual atomic packing clusters. However, these measurements did not address the evolution of the structure during plastic deformation. The objective of the present paper is to combine advanced TEM and rate sensitivity analysis in order to experimentally uncover the fundamental atomistic mechanisms responsible for the remarkable mechanical properties of the Zr_65_Ni_35_ films. The conclusions of the study will go beyond the specific alloy under investigation to be potentially applicable to the more general class of MGs in which the control of heterogeneous structure and local bond flexibility can lead to superior mechanical properties.

**Figure 1:**
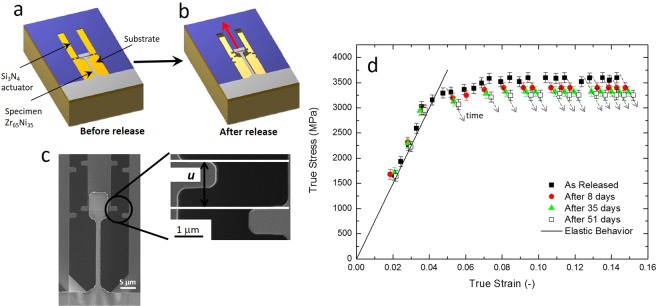
Lab-on-chip method used to determine the stress-strain response and the rate dependent behaviour. (**a**,**b**) schematic of the lab-on-chip before and after release, respectively. (**c**) SEM image of a deformed freestanding specimen. (**d**) True stress - true strain curves obtained using 25 μm × 1 μm × 360 nm specimens, highlighting the relaxation behaviour for different time interval up to 51 days after release.

Zr_65_Ni_35_ (% at.) TMGFs with a thickness of 360 nm were deposited by DC-magnetron sputtering^40,41^. Their composition has been verified by energy dispersive X-ray spectroscopy (EDS) and with electron probe micro analysis (EPMA) with no significant spatial variation^40,41^. The elastic properties were determined by Brillouin spectroscopy^40^ giving a Young’s modulus E = 72 ± 2 GPa and Poisson ratio *ν* = 0.40 ± 0.05. The amorphous structure was confirmed by X-ray diffraction (XRD) and TEM^40^. The films have been deformed using an on-chip test method based on a residual stress actuation principle. This technique involves lithography to produce the test specimens^19^. More precisely, long dogbone metallic glass specimens overlap with Si_3_N_4_ actuator beams involving tensile residual stress ~1 GPa^19^. The “actuator + specimen” constitutes one elementary test structure (Fig. 1a). Upon release by chemical etching of the underlying layer (Fig. 1b), the actuator contracts and pulls on the test specimen until force equilibrium is reached. One specimen corresponds to one point in the stress-strain curve. The test specimen is almost perfectly aligned with the loading direction. Such tests are simultaneously performed on hundreds of specimens with different dimensions in order to determine the stress-strain curve up to fracture^19^. Each specimen being deformed up to a specific strain and then releasing with time. A lower bound for the ductility is provided by the strain attained with the last unbroken specimen^19^. A Scanning Electron Microscopy (SEM) image after release is provided in Fig. 1c, showing the deformation of a 25 μm × 1 μm × 360 nm ZrNi uniaxial tension specimen. Figure 1d shows the stress-strain response determined based on 25 μm × 1 μm × 360 nm specimens. The curve is based on more than 20 specimens showing the reproducibility of the behaviour. The first measurements (black squares) made 2 hours after the release of the films show a large yield strength (~3250 MPa)/ductility (14.5%) balance^19^, in significant contrast with the brittle behaviour of most BMGs and many TMGFs reported in the literature. SEM as well as low magnification and aberration corrected high resolution TEM (HRTEM) investigations confirmed the absence of catastrophic shear bands^19^. The outstanding strength/ ductility balance exhibited by the films of Fig. 1c motivated further investigation of the elementary plasticity mechanisms.

In addition to the static response, the UCLouvain on-chip method can be directly used to determine the rate dependent behaviour of film specimens^42,43^. Indeed, once a stable position is reached for a particular test structure (Fig. 1b) in the force-displacement diagram, stress relaxation takes place with a kinetics depending on the thermally activated deformation mechanisms. The time domain that can be explored is potentially very large (i.e. here strain rates from 10^−7^ down to 10^−9^ s^−1^ are investigated) which correspond to measurements made up to 51 days after release in the present study, see Fig. 1d. These data contrast thus with respect to earlier viscoplastic analysis carried out by nanoindentation in which the characteristic strain rate is of the order of 10^−1^–10^−2^ s^−1^ ^40^ (knowing that applying lower strain rates with nanoindentation involves complex drift phenomena that are difficult to properly correct). The rate dependent measurements extracted from the on-chip method in Fig. 1d are very difficult to perform with compression test of micropillars or *in-situ* TEM tensile test^16,18,44–46^. Indeed, in the on-chip method, the specimens stay attached to the actuator leading to stress relaxation over long periods of time without the need of using any other test instruments. To the best of our knowledge, no similar techniques has been used to investigate the time dependent properties of micro-scale metallic glass specimens. Furthermore, taking advantage from the exceptional high plastic deformation levels attained in the present work, advanced TEM techniques including aberration corrected TEM imaging and spectroscopy as well as NBED have been used to investigate the nanoscale plasticity mechanisms. More technical details regarding the on-chip rate dependent measurements and TEM characterizations can be found in the Methods section.

## Results and Discussion

Figure 1d shows the deformation behaviour of the films for different time intervals equal to 8, 35 and 51 days, indicating significant creep/relaxation. Stress relaxation takes place only for stress levels above the yield stress. Only very small scatter is observed in the elastic regime (mainly due to statistical errors when measuring the displacement in the SEM). This is an interesting finding per se, meaning that there is no relaxation taking place below the static yield stress. The static yield stress is thus a threshold stress for generating plasticity at both moderate and very slow strain rates. The estimated activation volume (extracted from specimens releasing under stress larger than static yield stress) lies between 100 and 200 Å^3^ and is associated to a strain rate sensitivity exponent around 0.03 (see Methods and Supplementary Information for more details). This level of rate sensitivity is on the high range for metallic glasses^47^. These values agree with the results of earlier nanoindentation tests reporting an apparent activation volume V_act_ ~120–130 Å^3^, involving a different loading configuration and significantly larger strain rates^40^. All these evidences indicate that the deformation mechanisms remain similar over strain rates varying from 0.05 s^−1^ ^40^ down to 10^−9^ s^−1^, from classical plasticity regime to typical creep rates, consolidating the previous comment about the absence of creep below the static yield strength. According to Pan *et al*.^47^, the activation volume can be related to the average size of STZs considering a correction factor C′ equal to ~10. The present results thus correspond to an average size of STZs in between 1 and 2 nm^3^. A similar size of STZs was reported also by Kim *et al*.^48^ considering a Poisson ratio equal ν = 0.4 as well as by Yang *et al*.^49^ reporting an average size of STZs of ~2 nm for Zr_70_Ni_30_ (% at.) TMGFs. Moreover, in the present work, even after 51 days, both SEM and high resolution TEM analyses revealed that the deformation is homogeneously distributed within the specimens with no evidence of catastrophic shear bands^19^. The results extracted from the on-chip rate dependent analysis revealing a homogenous deformation process, a high rate sensitivity and an estimated value for the STZ size (1–2 nm^3^) motivated a deep TEM analysis of the underlying phenomena with the goal to unravel the atomic-scale plasticity mechanisms.

**Figure 2:**
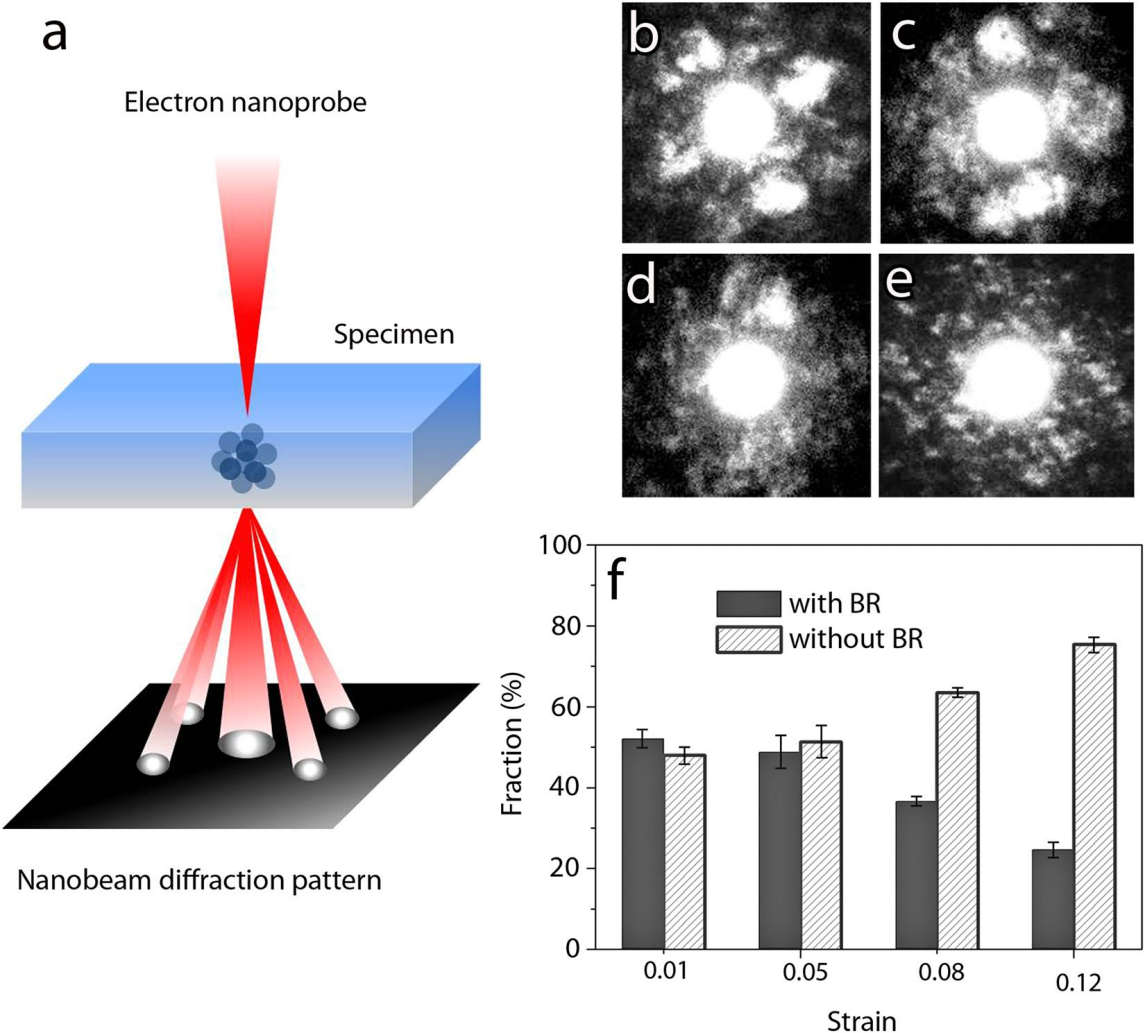
NBED technique and results on the 360 nm-thick Zr_65_Ni_35_ (% at.) films. (**a**) Principle of the NBED technique used to probe nanosized ordered regions in metallic glasses. Convergence angle α = 3.8 mrad and an electron beam with diameter of 0.4 nm have been used. (**b**–**d**) NBED patterns with strong Bragg reflections “BR” obtained on atomic clusters aligned in on-axis, three-beam and two-beam condition, respectively. (**e**) NBED pattern without BR. (**f**) Statistical evolution of the fraction of NBED patterns with and without BR obtained on the Zr_65_Ni_35_ thin films. 3400 NBED patterns have been acquired in regions with similar thickness as revealed by EELS (see Supplementary Information).

**Figure 3:**
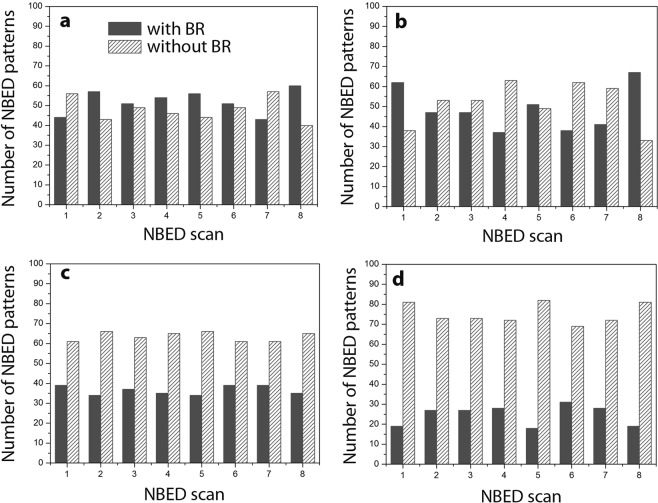
Number of NBED patterns with and without Bragg reflections in all the scanned regions for samples deformed to (**a**) 1%, (**b**) 5%, (**c**) 8% and (**d**) 12%.

Due to the amorphous nature of the microstructure, the activation of STZs could not be detected by conventional TEM nor by Cs corrected HR(Scanning)TEM. Thus, the NBED method was adopted in order to study the evolution of the local order with deformation. The measured spot dimension is in the range of the expected size of STZs. The basic principle of NBED is shown in Fig. 2a, consisting of a coherent electron beam with diameter of around 0.4 nm in order to produce two-dimensional diffraction patterns from atomic clusters with comparable size. Figure 2b–e show typical NBED patterns observed in Zr_65_Ni_35_ TMGFs. In Fig. 2b, a diffraction pattern with a set of two-fold symmetric spots (i.e. Bragg reflections), analogous to the diffraction pattern of a single crystal can be detected. A strong Bragg scattering is the signature of a locally ordered region (i.e., atomic clusters) with appropriate on-axis orientation with respect to the incident electron beam. Figure 2c,d show NBED patterns obtained on atomic clusters aligned in three-beam and two-beam condition, respectively. In the NBED pattern of Fig. 2e, Bragg reflections are not observed showing only a diffuse background with speckles. This corresponds to the absence of local ordering in randomly packed regions or to the presence of atomic clusters with an orientation not appropriate to produce strong Bragg reflections^39^. The graph of Fig. 2f shows the effect of increasing deformation on the fraction of NBED patterns with and without Bragg reflections in Zr_65_Ni_35_ TMGFs deformed elastically up to 1% and plastically up to 5%, 8% and 12%. Figure 3 shows details of the measurements shown in Fig. 2f. Multiple scannings were performed on different regions of each specimen, entering within the error bars on the graph of Fig. 2f. Each NBED scan in Fig. 3 corresponds to a square area of 4 × 4 nm^2^ obtained using an electron beam with diameter of 0.4 nm and step size of 0.4 nm. This leads to 100 NBED patterns for each scanned region. Eight NBED scans (i.e., 800 NBED patterns) have been acquired for each of the four deformation levels leading to 3200 NBED patterns in total (Fig. 3). All the measurements have been performed on regions with thickness around 15 nm as revealed by electron energy loss spectroscopy (EELS), see Supplementary Information.

Figures 2f and 3 show that the fraction of NBED patterns with and without Bragg reflections in films deformed to 1% and 5% are almost similar. However, a clear decrease (resp. increase) of NBED patterns with (resp. without) Bragg reflections is measured in specimens deformed to 8% and then up to 12%. This confirms that the local order decreases with increasing plastic deformation. The absence of a clear variation of the NBED patterns with and without Bragg reflections in specimens deformed to 5% can probably be attributed to the very small fraction of the activated events as most of the deformation is still elastic at this stage (see Fig. 1d). The results shown in Fig. 3 confirm that the plastic deformation in the Zr_65_Ni_35_ TMGFs is homogenous, involving the progressive and cooperative shear motion of atomic clusters throughout the entire volume of the specimen without any sign of catastrophic or strong localization. The results also confirm the interest of applying the NBED method for tracking STZ activation in metallic glasses. To the best of our knowledge, such behaviour has never been reported before in the literature. However, another fundamental question remains at this stage: why the activation of the STZs does not lead to a percolating pattern through the local mechanical interactions and to catastrophic shear bands?

**Figure 4:**
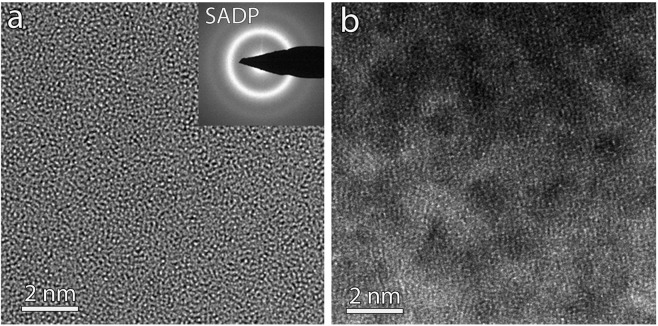
Aberration corrected HRTEM (**a**) and HAADF-STEM (**b**) obtained on as-deposited 360 nm-thick Zr_65_Ni_35_ film. Note the observation in (**b**) of nanoscale heterogeneities involving bright and dark nanodomains, not visible in (**a**).

**Figure 5:**
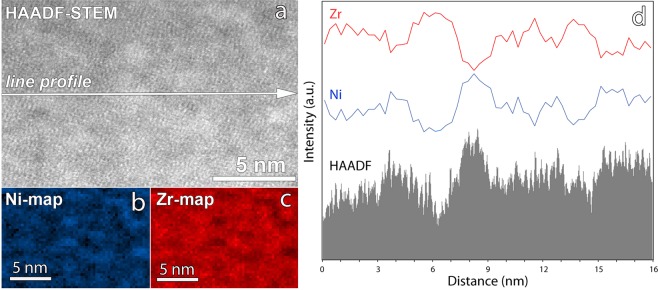
(**a**–**c**) HAADF-HRSTEM image and high resolution Ni and Zr chemical EELS maps obtained on as-deposited freestanding Zr_65_Ni_35_ metallic glass thin film, respectively. The EELS maps in (**b**,**c**) correspond to the HAADF-HRSTEM image in (**a**). (**d**) Intensity correlation between the HAADF-HRSTEM image and the EELS maps confirming the higher atomic density in the Ni-rich regions (Z_Ni_ < Z_Zr_).

Figure 4a,b exhibit Cs corrected HRTEM and high-angle-annular-dark-field high-resolution STEM (HAADF-HRSTEM) images of as-deposited Zr_65_Ni_35_ film, respectively. In the HRTEM image of Fig. 4a, only a homogenous maze-like pattern typical of an amorphous structure can be observed. No crystal-like ordered regions with lattice fringes are detected. Furthermore, the selected area electron diffraction (SAED) pattern shown in the inset of Fig. 4a exhibits a full halo ring with no detectable diffraction spots. All the Cs corrected HRTEM images and SAED patterns taken before and after deformation look similar. However, the HAADF-HRSTEM image of Fig. 4b clearly shows that the microstructure of the films is heterogeneous. Indeed, clusters in the form of bright and dark nanodomains can be observed. In Fig. 5, spatially resolved EELS reveals a very fine glassy nanostructure having well-defined Ni-rich clusters embedded in Zr-rich clusters with a characteristic length scale of ~2–3 nm. Furthermore, by correlating the EELS maps and the contrast in the HAADF-HRSTEM image in Fig. 5d, it can be seen that the Ni-rich clusters exhibit higher brightness compared to the Zr-rich clusters. As the HAADF-HRSTEM contrast is Z dependent, this observation contradicts the expectation related to the Z-contrast imaging mode since Z_Ni_ < Z_Zr_. To explain this result, one must invoke a higher (resp. lower) atomic density in the Ni-rich (resp. Zr-rich) clusters since the HAADF-STEM mode is very sensitive to atomic density variations. Based on these results, the following scenario is proposed to explain the absence of catastrophic shear bands as well as the remarkable strength/ductility balance exhibited by the ZrNi freestanding films used in this work.

Due to the high cooling rate upon deposition of the ZrNi TMGFs, the atomic arrangement might not reach an equilibrium packing state. The as-deposited film presumably exhibits a much looser atomic packing than the slowly quenched bulk counterpart, leading to a combination of dense nanosized Ni-rich clusters and Zr-rich clusters with lower atomic density (Fig. 5). Other side effects of the “sputtering process” could also play a role since recent MD simulations in the literature have shown that, in some specific cases, increasing the cooling rate could prohibit the formation of localized chemical heterogeneities^50^. During plastic deformation, defective zones (constituted by loosely packed atoms with excess free volume or increased capacity for bond switching (i.e. flexibility) act as preferential soft sites for the activation of STZs (or non-mature shear bands). Such defectives zones can be linked here to the less dense Zr-rich clusters and/or to the interfaces between the Zr-rich and the Ni-rich clusters, which might also confine excess free volume and higher flexibility. Note also that the average size of the compositional/density heterogeneities observed here is in agreement with the average size of STZs extracted from the time dependent mechanical tests shown in Fig. 1d (1–2 nm^3^). In the literature, TEM contrast of catastrophic shear bands is mainly attributed to slight changes of atomic density (from 1% to 10%) between sheared zones and the surrounding amorphous matrix^51^. Thus, non-mature nanoscale shear bands cannot be directly observed in high resolution TEM and STEM images (Fig. 4) because of the very small defect-size/foil thickness ratio. However, the activated defects cannot percolate to form catastrophic shear bands due to the presence of hard zones in the form of densely packed atomic Ni-rich clusters, leading to a more homogenous plastic deformation throughout the entire sample. This scenario is in agreement with the homogenous disruption of the local order revealed by NBED throughout the entire volume of the specimen (Fig. 3). Recently, Liu *et al*. studied Zr_55_Cu_30_Ni_5_Al_10_ sputter deposited metallic glass thin films using amplitude-modulation dynamic atomic force microscopy^23^. These authors revealed nanoscale viscoelastic heterogeneities with a characteristic length scale of 2–3 nm (very close to the length scale of the chemical/density heterogeneities shown in Fig. 5).

**Figure 6:**
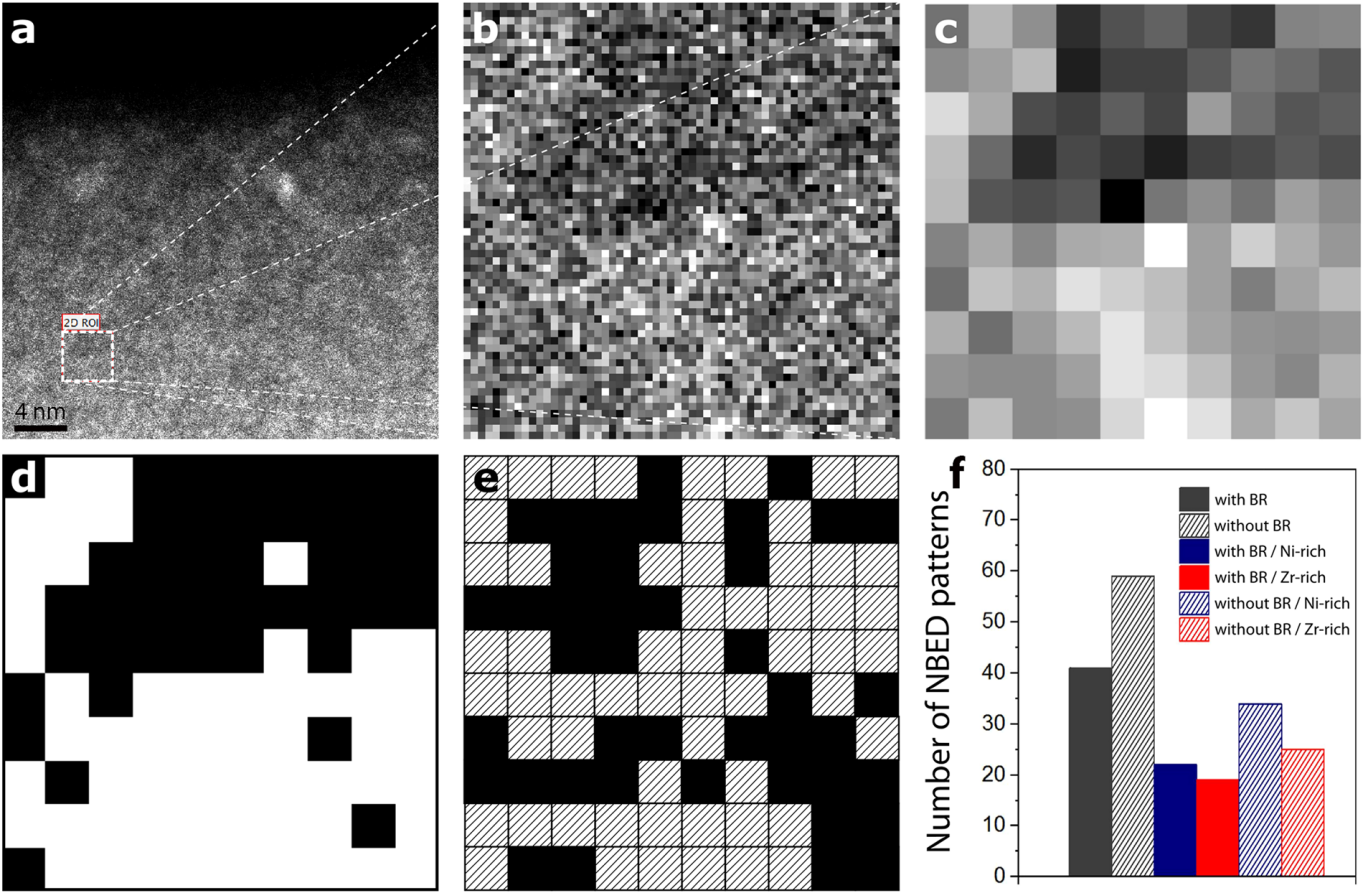
Example of NBED statistics on the Ni-rich and Zr-rich nanoclusters. (**a**) High resolution HAADF-STEM image from the as-deposited film. (**b**) Region selected for NBED scanning (4 × 4 nm^2^). It involves Ni-rich and Zr-rich nanoclusters. (**c**) Binning of the image in (**b**). (**d**) Binary image obtained by applying a virtual mask with a 50% threshold of grey level on (**c**). (**e**) NBED map obtained in (**b**). Black (resp. striped) squares correspond to NBED patterns with (resp. without) BR. (**f**) Number of NBED patterns with and without BR obtained from (**e**) as well as statistics generated by comparing (**d**,**e**) in order to include the role of the density/chemical heterogeneities. Colours in (**f**) are selected with reference to Figs 2f, 3 and 5(b,c).

**Figure 7:**
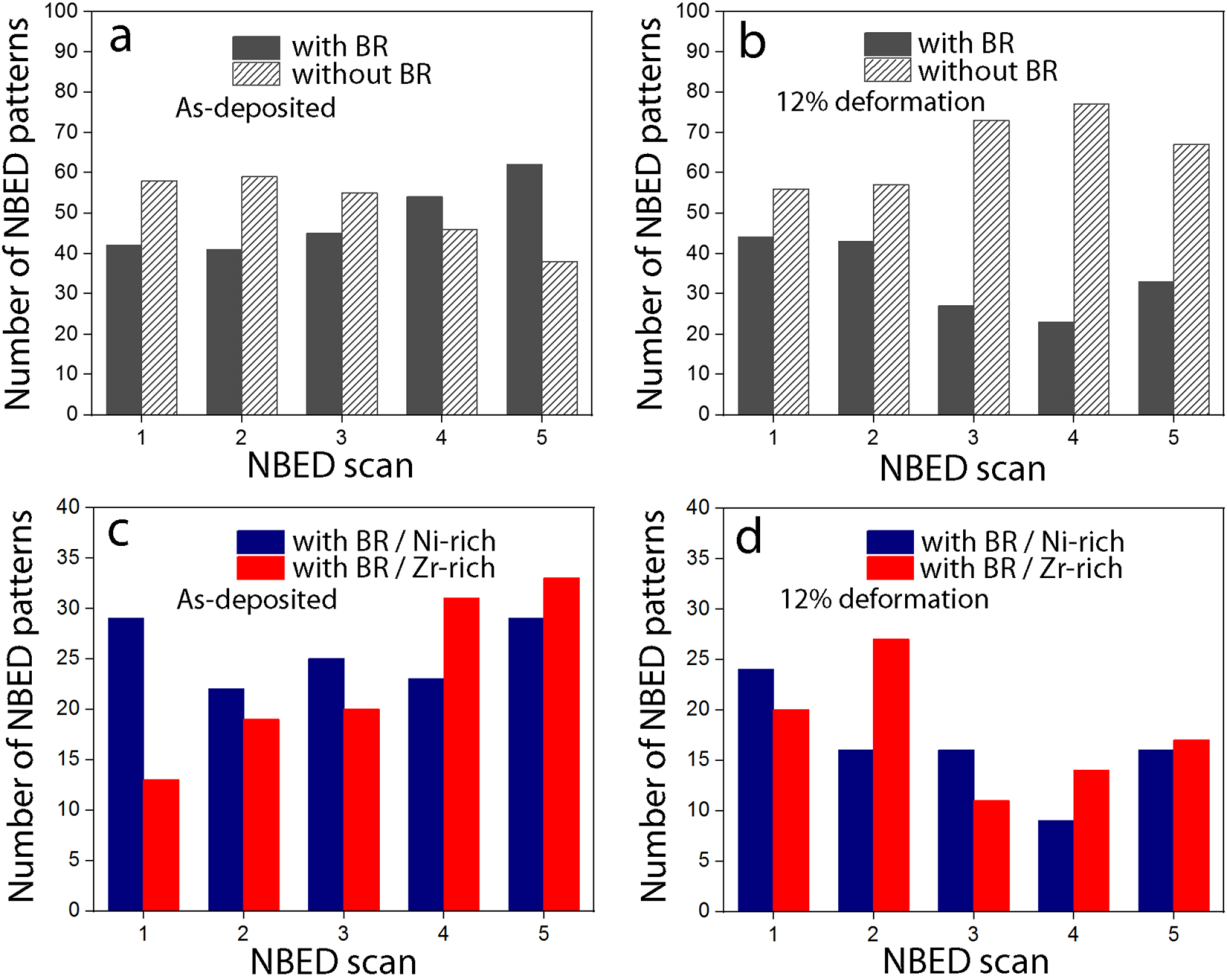
Number of NBED patterns with and without Bragg reflections in (**a**) as-deposited film and (**b**) film deformed to 12%. (**c**,**d**) Number of NBED patterns with Bragg reflections in the Ni-rich and Zr-rich clusters in as-deposited film (**c**) and in film deformed to 12%. (**d**) Colours are selected with reference to Fig. 6f.

In order to better understand the role of the density/chemical heterogeneities shown in Fig. 5 on the activation of the STZs, statistical NBED measurements have been performed on Zr-rich and Ni-rich clusters in as-deposited films and in films deformed up to 12%. First, the contrast and gamma factor of the HAADF µProbe-STEM images have been increased (Fig. 6a). NBED patterns were then acquired in a region involving a similar ratio of Zr-rich and Ni-rich clusters (Fig. 6b). The NBED acquisition parameters were identical to those used in the measurements shown in Fig. 3. Furthermore, in order to facilitate the tracking of the relationship between the density/chemical heterogeneities and the presence (or not) of atomic clusters, binning of the HAADF contrast of the NBED scanned region is applied in order to obtain a pixelated image (Fig. 6c). In this image, the total number of pixels is identical to the total number of NBED patterns acquired in the same region. This is followed by the application of a virtual mask with a 50% threshold of grey level in order to generate a binary image (Fig. 6d). This image is directly compared with the NBED map (Fig. 6e) in order to extract statistics on the number of NBED patterns with and without Bragg reflections on the Zr-rich and Ni-rich clusters (Fig. 6f). Figure 7 exhibits the results of this method in as-deposited film and in a film deformed to 12%. Ten NBED scans (i.e., 1000 NBED patterns) have been acquired for each film leading to 2000 NBED patterns in total. However, noticeable drift was observed after the acquisition of the first five NBED scans. Thus, Fig. 7 only exhibits NBED statistics obtained for the last five scans in which drift was excluded based on digital image correlation between before and after NBED maps acquisition. Three main comments can be made in Fig. 7: (i) Statistics on the number of NBED patterns with and without Bragg reflections obtained in the as-deposited films (Fig. 7a) are very similar to those obtained in the film elastically deformed to 1% (Fig. 3a). Again, this confirms that most of the deformation is still elastic at this stage. (ii) There is no clear relationship between the density/chemical heterogeneities and the number of NBED patterns with Bragg reflections in the as-deposited film (Fig. 7c), indicating the presence of local atomic order in both Ni-rich and Zr-rich clusters. (iii) Although the global disruption of the local order with increasing plastic deformation shown in Figs 2f and 3 (‘random’ NBED acquisitions) can be confirmed in film plastically deformed to 12% (Fig. 7b), no clear relationship between such behaviour and the density/chemical heterogeneities can be detected (Fig. 7d). This can be intuitively explained by assuming that an important fraction of STZs can be activated by shear along the interfaces between the Zr-rich and Ni-rich clusters. However, probing individual interfaces with NBED cannot be achieved in a TEM thin foil involving 3D spinodal-like nanoscale networks of density/chemical fluctuations with randomly oriented non-sharp interfaces. Very recently, Kim *et al*. confirmed the key importance of similar interfaces for increasing the resistance against shear band propagation in Cu-Zr-Al-Y bulk MGs^52^. Note in Fig. 7b that, in contrast with the scanned regions “3, 4, 5”, the fraction of NBED patterns with/without Bragg reflections in the scanned regions “1, 2” is very similar to the undeformed samples. This can be explained by the fact that no significant shear occurred along the selected Zr-rich/Ni-rich interfaces due to local changes of the structure of these interfaces.

The scenario emerging from the present investigation is very similar to ideas formulated in the context of ‘metallic nanoglasses (NGs)’^53–55^. The microstructure of this new class of MGs consists of nanometer-sized glassy grains separated by glass-glass interfaces exhibiting lower atomic density^53–55^ that can be partly assimilated to the microstructure of the present ZrNi films. Interestingly, NGs exhibits a transition in the deformation mechanism from a single mature shear band to homogeneous superplastic flow with decreasing average glassy grain size^30,56–58^. This was attributed to the high fraction of the soft glass-glass interfaces that can act as preferred sites for the formation of numerous ‘non-mature’ shear bands. However, in contrast with our ZrNi films, most of the studies in the literature have shown that the extraordinary elongation-to-failure observed in metallic nanoglasses is generally associated with pronounced drop of the strength (up to 60% for grain size <5 nm^57^) due to the presence of a significant volume fraction of soft glass-glass interfaces. This can be attributed to the fact that these simulations did not take into account the complexity of the ‘real’ microstructure including not only local variation of atomic density but also chemical and atomic order heterogeneities. Indeed, some recent experimental works in the literature reported that the microstructure of metallic nanoglasses can be much more complex, involving structurally and compositionally distinct, nanodispersed, glassy phases separated by sharp inter-phase boundaries (i.e., composite nanoglass)^59^. In the present study, the ZrNi films exhibit spinodal-like nanoscale networks of density/chemical fluctuations without pronounced sharp interfaces (Figs 4 and 5). Furthermore, the present ZrNi films exhibit a strength near the ideal value and a moderate strain hardening capacity^19^.

## Conclusion

The elementary plasticity mechanisms in freestanding Zr_65_Ni_35_ (% at.) TMGFs exhibiting outstanding strength/ductility balance have been investigated using advanced TEM characterization methods. The time dependence test carried out with a micro-actuated tensile technique has revealed that only plastically deformed specimens undergo stress relaxation. Moreover, the calculated activation volume is on the order of 100–200 Å^3^ providing an average size of STZs in the range 1 to 2 nm^3^. Statistical measurements using randomly acquired NBED patterns have shown progressive homogenous disruption of the local order with increasing plastic deformations due to the activation of STZ events throughout the entire volume of the films. This is also in agreement with the high deformability and the absence of catastrophic shear banding. High resolution HAADF-STEM and spatially resolved EELS revealed that the microstructure is heterogeneous involving dense Ni-rich clusters embedded in Zr-rich clusters with lower atomic density and a characteristic length scale of ~2–3 nm in agreement with the activation volume extracted from the rate dependence study. The heterogeneities form a 3D spinodal-like nanoscale networks of density/chemical fluctuations with randomly oriented non-sharp interfaces. The complex network made by these interfaces is expected to play a pivotal role for mediating the local shear excursion and for the increase of the resistance to plastic localization by enhancing the strain rate sensitivity and the ability for the delocalization of the plastic deformation. These findings highlight the key importance, beyond the sample dimensions, of accurate 3D quantitative measurements of the structural/chemical heterogeneities originating from the processing of TMGFs. On-going experiments are performed in order to investigate the evolution of the local order using NBED in deformed TMGFs with well-controlled density/chemical heterogeneities arranged in nanolayered structure. This will facilitate the probing of single glassy phases and interfaces in NBED experiments. Furthermore, similar characterizations will be performed on films with different thicknesses in order to investigate the influence of the initial microstructure on the size effects reported in the work of Ghidelli *et al*.^19^. The experimental approach used in the present study opens avenues to investigate the atomic-scale plasticity mechanisms in other classes of MGs with different structural heterogeneities and mechanical behaviour.

## Methods

In the UCLouvain on-chip method, the strain-rate sensitivity is evaluated from the stress versus strain evolution following the approach described in^42,43^ in order to provide a more quantitative insight into the relaxation mechanisms. The strain-rate-sensitivity exponent *m* is defined as $m=\frac{\partial ln\sigma }{\partial ln{\dot{\varepsilon }}_{p}}$ and the apparent activation volume *V* is defined as $V=M{k}_{B}T\frac{\partial ln{\dot{\varepsilon }}_{p}}{\partial {\sigma }_{th}}$, where *σ*_*th*_ is the thermally activated contribution to the stress, ${\dot{\varepsilon }}_{p}$ is the plastic strain rate, *M* is the Taylor factor, *k*_*B*_ is the Boltzmann constant and *T* is the temperature. However, as a first simple means to analyze the displacements *u* versus time *t* data, a logarithmic variation is assumed in the form *u* = *A*_1_ ln (*A*_2_*t*) where *A*_*1*_ and *A*_*2*_ are fitting parameters^60^. Imposing a logarithmic variation of *u* with *t* leads to the extraction of a constant mean strain rate sensitivity independent of time. (see Supplementary Information for more details).

Regarding TEM characterizations, high resolution (scanning) TEM imaging as well as spatially resolved EELS were performed on a FEI Titan “cubed” microscope, operated at a 200 kV acceleration voltage and equipped with both TEM and STEM aberration correctors. Aberration-corrected TEM imaging was performed under negative Cs conditions (Cs tuned to −12 μm). HAADF-STEM images were acquired using a convergence semi-angle α of 22 mrad and 25 pA probe current, the ADF inner acceptance angle β and the EELS acceptance angle were both equal to 28 mrad. Quantification of the Zr and Ni content was performed with the Gatan Digital Micrograph (DM) software package, using Hartree–Slater cross sections. NBED patterns were acquired on a FEI Titan transmission electron microscope, operating at an accelerating voltage of 200 kV, equipped with a probe Cs corrector and a Gatan US1000XP CCD camera. The low convergence angle electron beam (α = 3.8 mrad) was obtained by using the µProbe STEM mode with a 20 µm C2 condenser aperture. The presence of an independent C3 lens in the microscope allowed having flexibility in the value of the convergence angle. In µProbe STEM, the probe corrector is optically inactive but was tuned in standard STEM mode prior to the experiment to ensure low aberration values. Cross-sectional TEM foils were prepared using FIB from as-deposited films and from films deformed to 1%, 5%, 8% and 12% using the lift out method in a FEI dual beam FIB/SEM instrument. A protective platinum layer using electron beam assisted deposition has been used. An ion beam of 2 kV/0.2 nA was employed to achieve the final thinning and to minimize defects generated during high voltage FIB thinning on both sides of the sample.

## Supplementary information

Supporting Information

## Data Availability

Raw data are available upon request.
