# Supplementary material for: Atomic-scale viscoplasticity mechanisms revealed in high ductility metallic glass films
Source: Sci Rep. 2019 Sep 17;9:13426. doi: 10.1038/s41598-019-49910-7 (PMC6749058; doi:10.1038/s41598-019-49910-7)
Supplement: Supplementary file 1 — Supporting Information [file 41598_2019_49910_MOESM1_ESM.pdf]

Supplementary Information

Atomic-scale mechanisms of viscoplasticity revealed in high ductility metallic glass films

Hosni Idrissi[1,2]*, Matteo Ghidelli[3,1,4], Armand Béché[2], Stuart Turner[2], Sébastien Gravier[3], Jean-Jacques Blandin[3], Jean-Pierre Raskin[4], Dominique Schryvers[2], Thomas Pardoen[1]

[1] Institute of Mechanics, Materials and Civil Engineering, UCLouvain, B-1348, Louvain-la-Neuve, Belgium.
[2] EMAT, University of Antwerp, Groenenborgerlaan 171, B-2020 Antwerp, Belgium
[3] Science and engineering of materials and processes, SIMaP, Université de Grenoble / CNRS, UJF / Grenoble INP, BP46, 38402, Saint-Martin d'Hères, France.
[4] Institute of information and communication technologies, electronics and applied mathematics, ICTEAM, Université catholique de Louvain, B-1348, Louvain-la-Neuve, Belgium.

*The corresponding author:
hosni.idrissi@uclouvain.be

Analysis of the relaxation kinetics of on-chip specimens

Fig. S1 shows the evolution of the average displacement as a function of time for a set of test beams with the same dimensions: 25 µm x 1 µm x 360 nm. These dog bone beams have been pulled in tension owing to the contraction of actuators of different lengths, see Ref. 18 in the main text. The actuator width and thickness are equal to 15.25 µm and 160 nm, respectively, while the lengths varies between 100 and 1350 µm. The four different time intervals are respectively equal to 3 hours (as released sample), 7 days 21 days and 51 days. In Fig. S1, full symbols correspond to specimen beams that deform elastically without relaxation (Fig. 2 in the paper), while empty symbols correspond to plastically deformed specimens - relaxing the stress over time (Fig. 2 in the paper), corresponding to actuator lengths larger than ≥ 600 µm, see Fig. S2. For these samples a logarithmic fit has been applied with the equation $u = A_1 \ln(A_2\, t)$ (solid lines, Fig. S1) with a regression coefficient $R^2 > 0.988$. The average activation volume (details in the paper) can be extracted based on the coefficients A_1 and A_2.

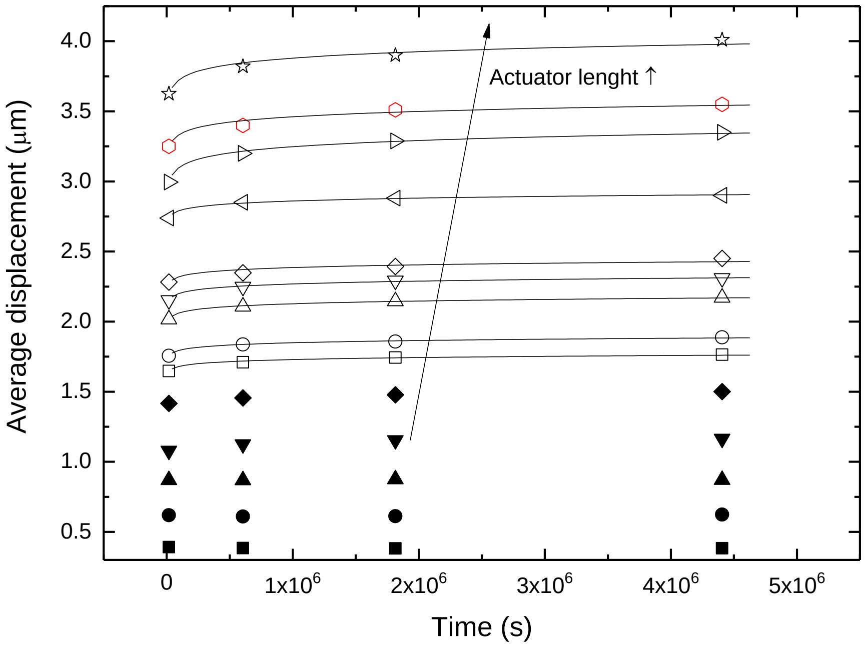

Figure S1. Average displacement vs time for a beam of 25 µm x 1 µm x 360 nm. Plastically deformed specimens (empty symbols) relax following a logarithmic kinetics as shown by the fitting lines.

Fig. S2 shows the variation of the average activation volume for a specimen beam of 25 µm x 1 µm x 360 nm as a function of the actuator length varying from 600 up to 1350 µm, corresponding to the empty symbols in Fig. S1. The activation volume is roughly constant ~130 $Å^3$, meaning that, although different stress and strain levels are involved, the underlying elementary deformation mechanisms controlling the relaxation remain the same.

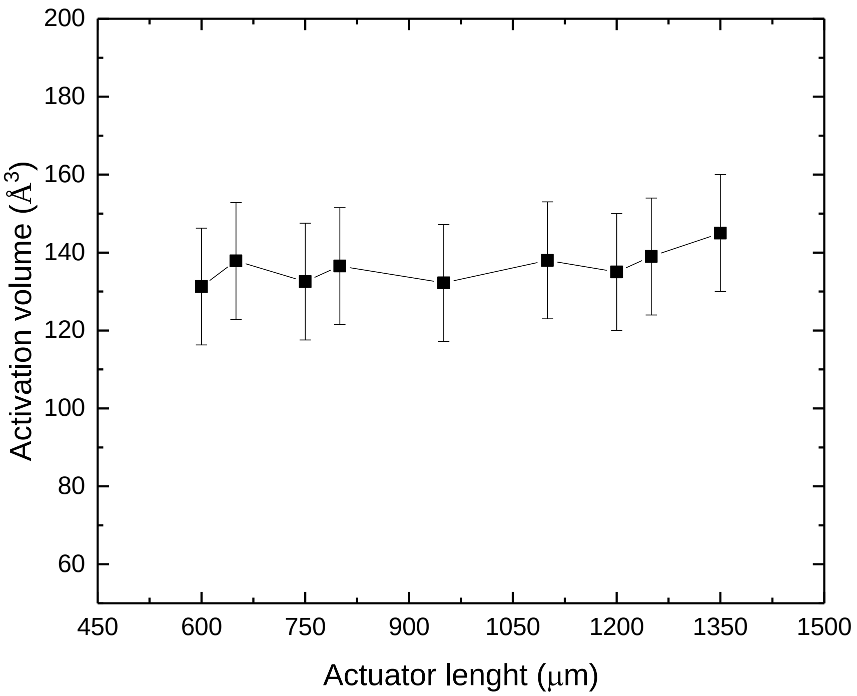

Figure S2. Average activation volume for different specimen beams as a function of the actuator length. The activation volume is constant ~130 Å^3.

Thickness maps by EELS

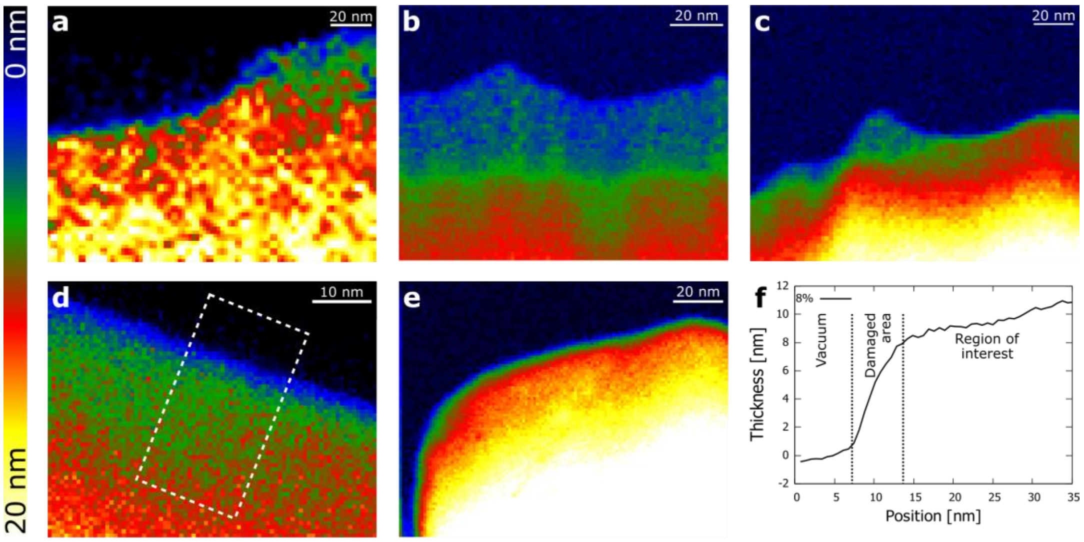

Figure S3. Thickness maps obtained with EELS in as-deposited films (**a**) as well as film deformed to (**b**) 1%, (**c**) 5%, (**d**) 8% and (**e**) 12%. (**f**) Example of thickness profile obtained from (**d**). All the NBED measurements shown in the main manuscript were performed in regions with thickness around 15 nm. Regions very close to the edge of the foil where FIB damages could dominate the microstructure were systematically avoided.
